# Diving into the proteomic atlas of SARS-CoV-2 infected cells

**DOI:** 10.1038/s41598-024-56328-3

**Published:** 2024-03-28

**Authors:** Victor C. Carregari, Guilherme Reis-de-Oliveira, Fernanda Crunfli, Bradley J. Smith, Gabriela Fabiano de Souza, Stéfanie Primon Muraro, Veronica M. Saia-Cereda, Pedro H. Vendramini, Paulo A. Baldasso, Lícia C. Silva-Costa, Giuliana S. Zuccoli, Caroline Brandão-Teles, André Antunes, Aline F. Valença, Gustavo G. Davanzo, João Victor Virgillio-da-Silva, Thiago dos Reis Araújo, Raphael Campos Guimarães, Felipe David Mendonça Chaim, Elinton Adami Chaim, Carolina Mie Kawagosi Onodera, Raissa Guimarães Ludwig, Tatiana Dandolini Saccon, André R. L. Damásio, Luiz Osório S. Leiria, Marco Aurélio R. Vinolo, Alessandro S. Farias, Pedro M. Moraes-Vieira, Marcelo A. Mori, José Luiz P. Módena, Daniel Martins-de-Souza

**Affiliations:** 1 Laboratory of Neuroproteomics, Department of Biochemistry and Tissue Biology, Institute of Biology, University of Campinas (UNICAMP), Campinas, São Paulo, Brazil; 2 Laboratory of Emerging Viruses, Department of Genetics, Evolution, Microbiology and Immunology, Institute of Biology, University of Campinas (UNICAMP), São Paulo, Brazil; 3 Department of Biochemistry and Tissue Biology, Institute of Biology, University of Campinas (UNICAMP), Campinas, São Paulo, Brazil; 4 Laboratory of Immunometabolism, Department of Genetics, Evolution, Microbiology and Immunology, Institute of Biology, University of Campinas (UNICAMP), São Paulo, Brazil; 5 Department of Pharmacology, Ribeirão Preto Medical School (FMRP), University of São Paulo (USP), Ribeirão Preto, São Paulo, Brazil; 6 Center for Research in Inflammatory Diseases, Ribeirão Preto, SP Brazil; 7 Obesity and Comorbidities Research Center (OCRC), Campinas, São Paulo, Brazil; 8 Department of Surgery, Faculty of Medical Sciences, University of Campinas, Campinas, SP Brazil; 9 Hematology-Hemotherapy Center, University of Campinas, Campinas, SP Brazil; 10 Laboratory of Immunoinflammation, Department of Genetics, Microbiology and Immunology, Institute of Biology, University of Campinas (UNICAMP), Campinas, São Paulo Brazil; 11 Instituto Nacional de Biomarcadores em Neuropsiquiatria (INBION), Conselho Nacional de Desenvolvimento Científico e Tecnológico, São Paulo, 05403-000 Brazil; 12 D’Or Institute for Research and Education (IDOR), São Paulo, 04501-000 Brazil; 13 Autoimmune Research Laboratory, Department of Genetics, Microbiology and Immunology, Institute of Biology, University of Campinas (UNICAMP), Campinas, São Paulo, Brazil; 14 Experimental Medicine Research Cluster (EMRC), University of Campinas (UNICAMP), Campinas, São Paulo, Brazil

**Keywords:** Proteins, Proteomics, Viral infection

## Abstract

The COVID-19 pandemic was initiated by the rapid spread of a SARS-CoV-2 strain. Though mainly classified as a respiratory disease, SARS-CoV-2 infects multiple tissues throughout the human body, leading to a wide range of symptoms in patients. To better understand how SARS-CoV-2 affects the proteome from cells with different ontologies, this work generated an infectome atlas of 9 cell models, including cells from brain, blood, digestive system, and adipocyte tissue. Our data shows that SARS-CoV-2 infection mainly trigger dysregulations on proteins related to cellular structure and energy metabolism. Despite these pivotal processes, heterogeneity of infection was also observed, highlighting many proteins and pathways uniquely dysregulated in one cell type or ontological group. These data have been made searchable online via a tool that will permit future submissions of proteomic data (https://reisdeoliveira.shinyapps.io/Infectome_App/) to enrich and expand this knowledgebase.

## Introduction

The COVID-19 pandemic, caused by the severe acute respiratory syndrome coronavirus 2 (SARS-CoV-2), has been responsible for more than 649·8 million confirmed cases and 6·6 million deaths as of December 2022^1^, drastically affecting the global economy and social behaviour. COVID-19 is manifested by pulmonary infection and a cytokine storm in more severe stages of the disease. However, a growing number of studies have also proven the presence of SARS-CoV-2 in various other tissues and biofluids, such as cardiac muscle tissue, the central nervous system (CNS), the kidneys, the gastrointestinal system, and adipose tissue^2–6^.

The genome of the SARS-CoV-2 encodes 29 proteins, including 16 non-structural, 4 structural and 9 accessory proteins (NPs, S, M, E, N and Accessory proteins). SARS-CoV-2 is able to infect human cells and tissues mainly through their expression of angiotensin-converting enzyme 2 (ACE2), the main port of entry for the virus^2,7,8^; however, other receptors have been described as secondary for viral infection, such as TPMRS22, NPR1, and BSG/CD147^9,10^ Once inside the cell, SARS-CoV-2 then alters cell function at the proteomic level at minimum, often resulting in tissue dysfunction and damage.

Xiu et al. performed a broad proteomic analysis in distinct post-mortem tissues from several organs obtained from patients who died of COVID-19^8^, in which they discovered distinct patterns of protein expression for each infected organ. To complement this study, an easily searchable compilation of general and tissue-specific molecular mechanisms that are triggered by the virus in different cell types is relevant. With this in mind, we analysed proteomic data from several distinct SARS-CoV-2-infected cell types: neural stem cell (NSC)-derived astrocytes and neurons^11^; SH-SY5Y (human neuroblastoma cells) and CACO-2 (intestinal epithelial cells) cultured cells; T-cells and monocytes isolated from human blood^12^; hepatocytes; and adipocytes isolated from visceral and subcutaneous tissue^13^.

This information brings new insight into how the virus disturbs biochemical processes and can therefore be used to generate new integrated therapeutic targets, which may be useful to treat COVID-19 systemically and in a tissue-specific manner. Moreover, with the creation of the online SARS-Cov-2 infectome, a new tool to research proteins of interest will become available for public browsing, also including how SARS-CoV-2 can affect these proteins in each cell type and which biological mechanisms are altered by the infection.

## Results

For this study, all cells analysed were infected “in vitro” with the SARS-CoV-2 virus with the same Multiplicity of Infection (MOI) promoting data homogeneity. Except for SH-SHY5 and HepG2 cells, all other cell lines used in this investigation originated from humans and underwent subsequent differentiation processes, as outlined in the methods section. Before any subsequent analyses, a viral kinetics analysis at three time points indicated that all cells analysed here were indeed infected by SARS-CoV-2. Data regarding T-cells, monocytes, adipocytes, and astrocytes were included in their originally published articles^11–13,28^. Despite being able to replicate within CNS cells, the virus was not able to secrete viral particles into the medium; all other cell types showed signs of both viral replication and secretion (Supplementary Fig. 1).

**Figure 1:**
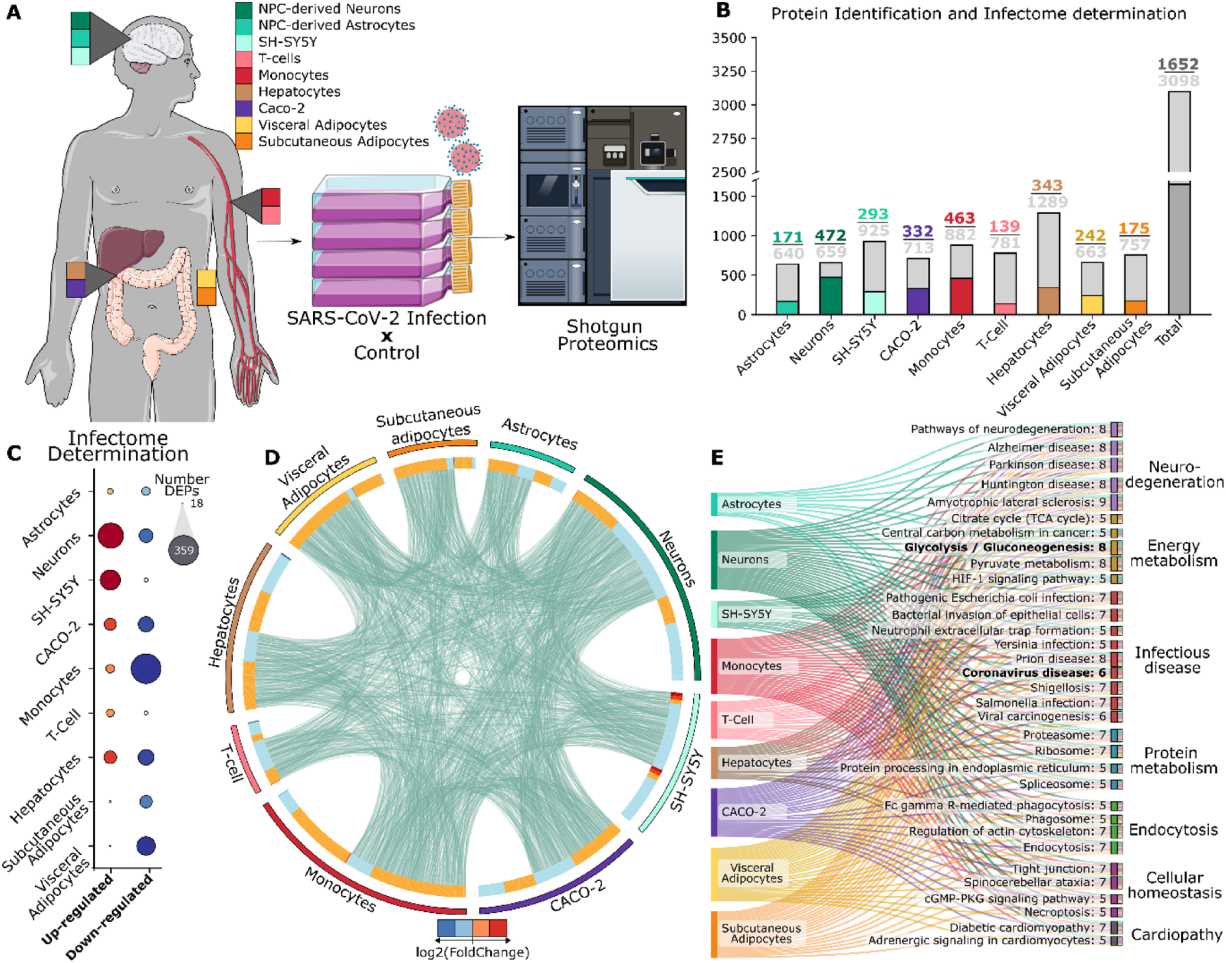
SARS-CoV-2 infects cells from different tissues and elicits changes in protein abundance. (**A**) Cell lines from distinct tissues and biological systems were infected with SARS-CoV-2 in vitro and the infectomes (proteins differentially regulated due to viral response) were obtained by shotgun proteomics. (**B**) Total number of identified proteins (gray) and differentially regulated proteins (colored) in each cell type. (**C**) Proteins differentially regulated in the respective infectomes separated by direction of regulation. Bubble size indicates the number of differentially expressed proteins (DEPs). (**D**) Overview of SARS-CoV-2 infectome in all cell types (outer circle), showing proteins that are shared among groups as well as their respective regulation in the heatmap (inner circle). (**E**) Sankey plot of pathways enriched in at least 50% of infectomes.

Proteomic analyses of SARS-CoV-2-infected cells were used to build what we will hereby refer to as the SARS-CoV-2 infectome, a compilation of differentially expressed proteins in cells in response to SARS-CoV-2 infection (Fig. 1A, and Supplementary Table 1). Among all analyses, a total of 3098 proteins were quantified, of which 1652 were found differentially regulated (*p*-value < 0·05, Fig. 1B). Our proteome analysis revealed a high level of data reproducibility and a wide range of identified protein abundance, as corroborated by dynamic range analysis (Supplementary Fig. 2 and 3). It is also noteworthy to emphasize that very few missing values were identified, and less than 2% of the peptides exhibited more than 2 missed cleavages.

Pair-wise Pearson’s correlation analysis, followed by hierarchical clustering, showed that almost all cell types clustered as expected by their ontology at the whole-proteome level, a pattern that was markedly distinct while considering only differentially regulated proteins (Supplementary Fig. 4). These differences may be explained by high diversity effects elicited by SARS-CoV-2 in each cell type, which can vary in the number of differentially regulated proteins as well as their regulation direction (Fig. 1C). Interestingly, neuronal cell models were unique in that they presented more upregulated proteins in their infectome. Additionally, we observed that 551 proteins were dysregulated in more than one cell type; however, none were found differentially regulated in all cell types (Fig. 1D, Supplementary Fig. 5).

A pathway enrichment analysis for each infectome identified 151 biological pathways associated with SARS-CoV-2 infection, with the neuronal infectome being the largest contributor (Supplementary Fig. 6). When filtering for pathways enriched in at least 50% of infectomes, SARS-CoV-2 modulated the protein related to energy metabolism (mainly glycolysis), infectious diseases, protein metabolism (both synthesis and degradation processes), signalling/homeostatic pathways, and potential comorbidities (Fig. 1E) in nearly all cells, in line with expected responses to viral infection and viral particle production. Six cellular infectomes (neurons, SH5Y, monocytes, hepatocytes, and both types of adipocytes) also enriched for Coronavirus disease, highlighting the canonical infection pathways in several cell types outside of the respiratory system. A protein–protein interaction network enriched for coronavirus disease reveals several key proteins ubiquitously present dysregulated across all the six cell types (Supplementary Fig. 7A). Analysis of the proteins associated with Coronavirus Disease reveals a deficiency of these pivotal proteins in Astrocytes, Caco-2, and T-Cells, as depicted in Supplementary Fig. 7B.

Given the variety of cellular functions and complexity of the respective infectomes of each cell type, we categorised differential protein expression by ontology: CNS cells (neurons, differentiated SH-SY5Y neuron-like cells, and astrocytes), gastroenterological cells (CACO-2, hepatocytes), white blood cells (monocytes and T-cells), and adipose tissue (differentiated visceral and subcutaneous adipocytes). This allowed an evaluation of similarity among cellular infectomes within ontological groups, useful in increasing our understanding about changes triggered by SARS-CoV-2 to better predict body response to infection.

All results and findings derived from these proteomic analyses were compiled and published in a unique database, forming the SARS-CoV-2 Infectome Atlas. This atlas, being the first of its kind, details the proteomic dysregulations elicited by SARS-CoV-2 in nine cellular infectomes, groupable by their tissue ontology. Individual proteins are searchable, providing information about normalised fold change differences, as well as altered pathways and their constituent proteins. This allows for targeted searching of proteins and pathways of interest within a SARS-CoV-2 context (https://reisdeoliveira.shinyapps.io/Infectome_App/).

### SARS-CoV-2 infection modulates CNS cell proteomes

**Figure 2:**
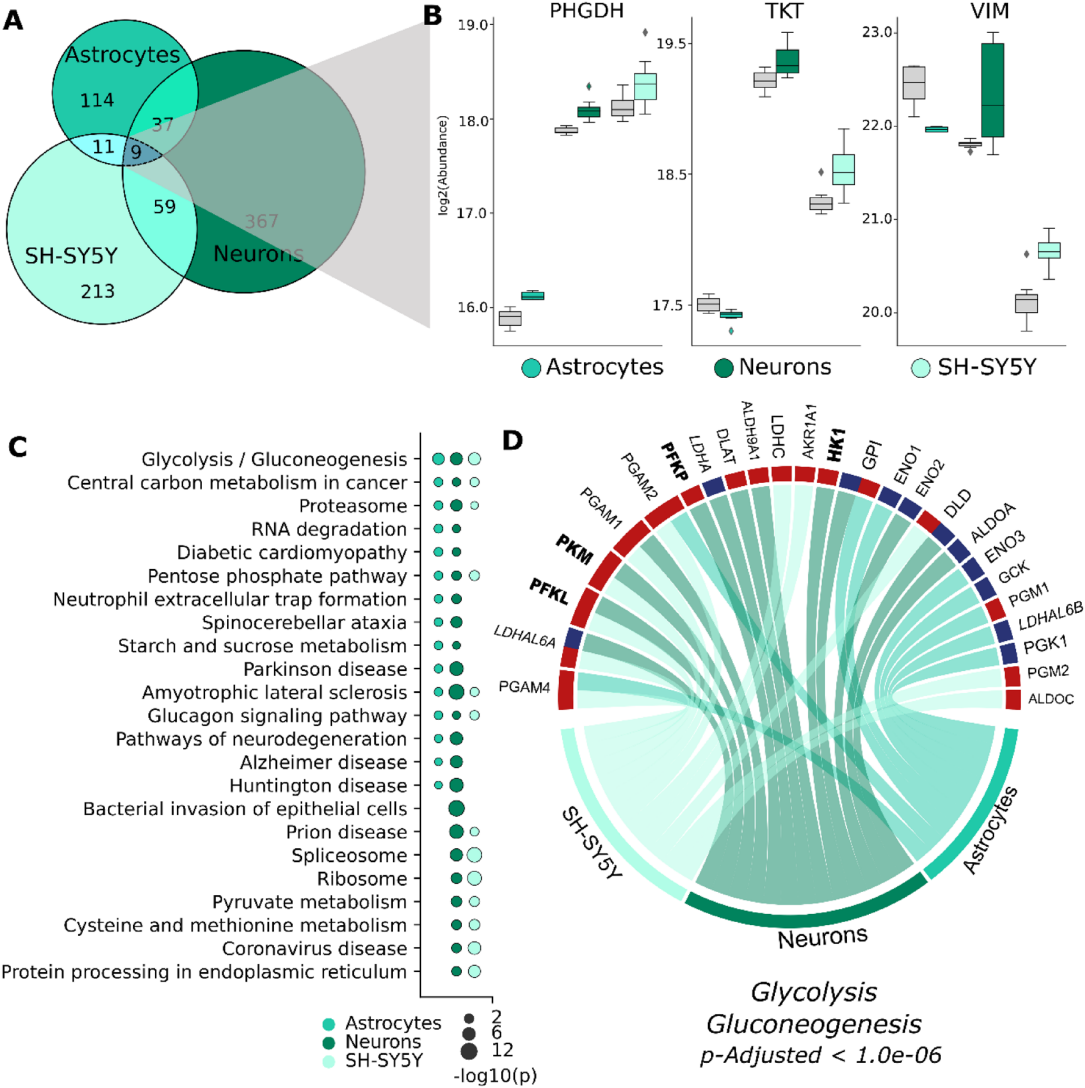
SARS-CoV-2 induces changes in the proteomes of CNS cell types. (**A**) Venn diagram showing differentially expressed proteins among NPC-derived astrocytes and neurons and differentiated SH-SY5Y neurons. (**B**) Normalized abundance of the differentially regulated proteins associated with energy metabolism (PHGDH, TKT) and cellular marker (VIM) for CNS cells. Gray bars represent the abundance in uninfected cells. (**C**) Pathway enrichment analyses for each CNS infectome. The top 10 pathways were selected for each cellular model, and then a pair-wise comparison was performed to visualize enrichment in other cell types. Bubble size indicates adjusted *p*-value on a − log10 scale. (**D**) Upregulated (red) and downregulated (blue) proteins associated with glycolysis in different CNS cell types. Proteins in bold and italic are pivotal in glycolysis and lactate generation, respectively.

The infectome of CNS cells was composed of 810 differentially regulated proteins, with astrocytes presenting the fewest dysregulations (Fig. 2A). Nine differentially regulated proteins were observed in all cell types, primarily associated with translation machinery (RPL29, RPL22, PABPC1, RPL12) and energy metabolism (TKT, PHGDH). Interestingly, however, these proteins presented a distinct regulation profile in each CNS cell, despite similarities in function and role.

In energy metabolism, for instance, PHGDH, a pivotal protein throughout neurodevelopment, was upregulated in all infected cell types; whereas TKT was upregulated in both neuronal cell types but downregulated in astrocytes (Fig. 2B). Astrocytic marker vimentin (VIM), also present in excitatory neurons, was downregulated in infected astrocytes, and upregulated in both infected neuronal cell types (Fig. 2B). This divergent regulation pattern indicates that, even when cells share proximal ontological characteristics, SARS-CoV-2 can induce different and even divergent protein-level responses.

To understand how SARS-CoV-2 can potentially affect these in vitro CNS models at the functional level, the Kyoto Encyclopedia for Genes and Genomes (KEGG) was used for in silico enrichment analyses^32^. Performing a pair-wise comparison of the top ten pathways enriched for each cellular model (Fig. 2C), the NPC-derived neuron infectome was the only CNS cell type seen to incorporate all of the top ten terms found in the other CNS cell types. When both neuronal infectomes were compared, differentiated SH-SY5Y cells enriched 67% fewer terms than NPC-derived neurons, highlighting that SARS-CoV-2 response can differ by cell model, even of the same cell type (Supplementary Fig. 6). Nevertheless, at the pathway level, these cellular infectomes still shared several enriched pathways, such as glycolysis, pentose phosphate pathway, and proteasomes (Fig. 2C).

Since glycolysis was found enriched among the top ten enriched terms of all CNS infectomes (*p*-adj. < 1·0e-6), we highlighted the proteins differentially regulated in this pathway in each cell type (Fig. 2D). Most of the proteins (66%) associated with glycolysis were upregulated. In neuronal models, the proteins that play pivotal roles in glycolytic regulation (hexokinase [HK1], phosphofructokinase [PFKP/PFKL], and pyruvate kinase [PKM]) were upregulated after infection (Fig. 2D, bold); while in NSC-derived cells, lactate dehydrogenase-associated LDHAL6B was down-regulated. Additionally, PGM2 and PGM4 were upregulated in SH-SY5Y cells and NSC-derived astrocytes, both of which are essential in 5′-phosphopentose metabolism and nucleotide synthesis.

### SARS-CoV-2 infection modulates gastroenterological cell proteomes

**Figure 3:**
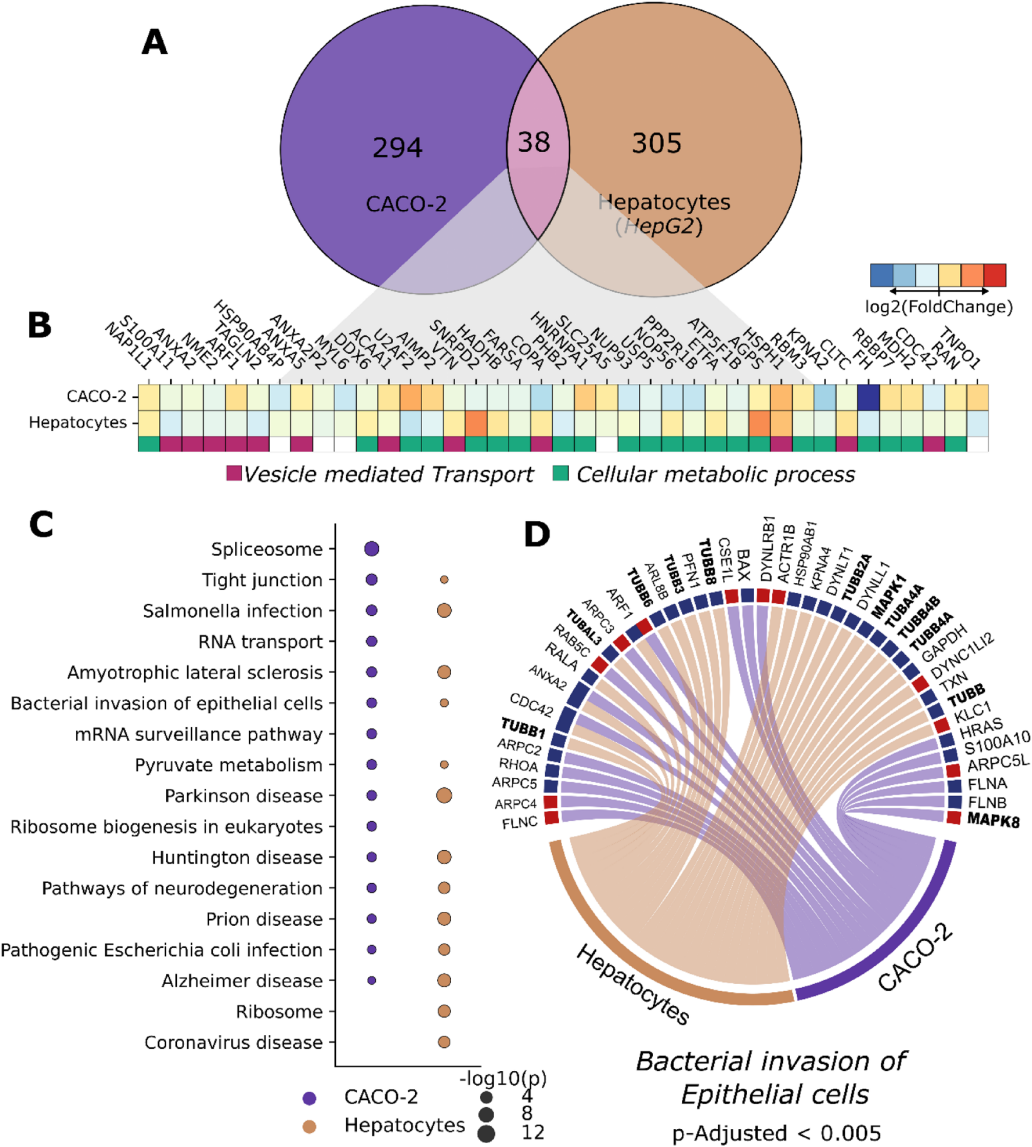
SARS-CoV-2 induces changes in the proteomes of gastroenterological cell types. (**A**) Venn diagram showing differentially expressed proteins in HepG2 and CACO-2 cells. (**B**) Heatmap of fold changes for each protein found in both gastroenterological cell types. (**C**) Pathway enrichment analyses for hepatocytes and CACO-2 cells. Bubble size indicates adjusted *p*-value on a − log10 scale. (**D**) Upregulated (red) and downregulated (blue) proteins related to bacterial invasion of epithelial cells. Proteins highlighted in bold are from the MAPK and tubulin families.

Proteome changes were evaluated in intestinal epithelial cells (CACO-2 cell line) and hepatoma cells (HepG2 cell line), both of which derive from cancer patients and present epithelial-like morphology. Infected CACO-2 cells presented 332 deregulated proteins while HepG2 cells presented 343 deregulated proteins; 38 proteins were deregulated in both cell lines (Fig. 3A). These overlapping proteins are related to vesicle-mediated transport (import into the nucleus, endocytosis, and exocytosis) and cellular metabolic processes (Fig. 3B).

Of the terms obtained from KEGG enrichment, 11 pathways were shared between both gastroenterological cell lines (Fig. 3C). Among the enriched terms, we found bacterial invasion of epithelial cells, Salmonella infection, and pathogenic *E. coli* infection. These processes could be associated with the diarrhoea induced by SARS-CoV-2 infection through a mechanism similar to previously documented bacterial invasion processes in the gut. Among the proteins associated with these pathways, only CDC42, ANXA2 and ARF1 were differentially regulated in both cell types; nevertheless, protein families (such as tubulin or MAPK), are shared in both cell types (Fig. 3D), which could lead to similar effects on epithelial homeostasis.

### SARS-CoV-2 infection modulates immune cell proteomes

**Figure 4:**
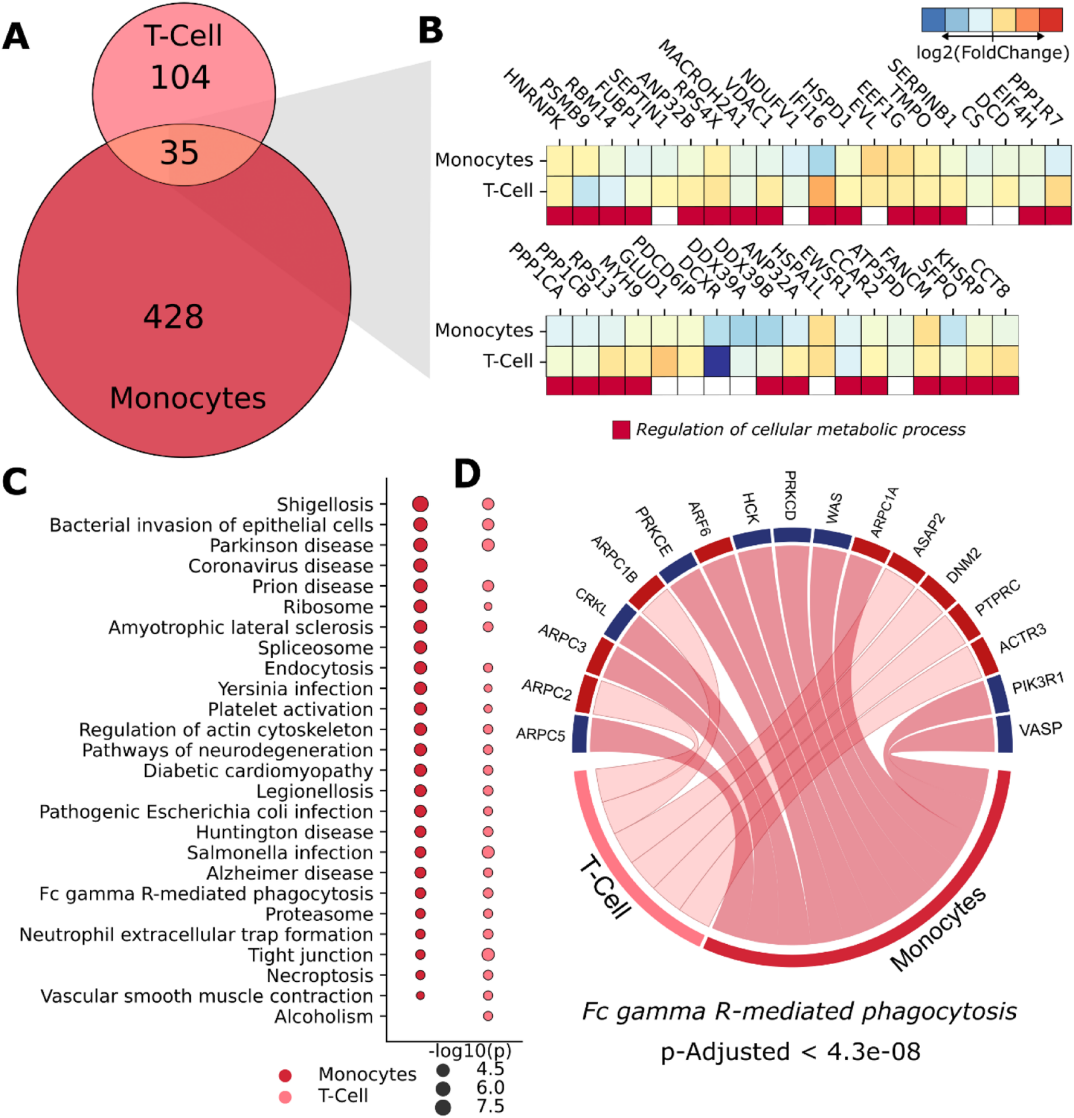
SARS-CoV-2 induces changes in the proteomes of immunological cells. (**A**) Venn diagram showing differentially expressed proteins in monocytes and lymphocytes. (**B**) Heatmap of fold changes for proteins found in both immune cells. (**C**) Pair-wise comparison of top pathways enriched against the KEGG database. Bubble size indicates adjusted *p*-value on a − log10 scale. (**D**) Upregulated (red) and downregulated (blue) proteins involved in Fc gamma R-mediated phagocytosis.

The immunological cells analysed in our current datasets consist of SARS-CoV-2-infected primary monocytes and T lymphocytes. By testing these two cell types, both myeloid and lymphoid lineages can be tested, covering the innate immune response with monocytes and the adaptive response with the lymphocytes. Our quantitative analysis found 139 proteins dysregulated in T-cells and 463 in monocytes, with 35 proteins dysregulated in both cell types (Fig. 4A). These proteins are associated with cellular metabolic processes; though the heatmap shows that fold change—and therefore likely the cellular response as well—can vary between myeloid and lymphoid cells (Fig. 4B).

Pathways enriched by the monocyte and lymphocyte infectomes (Fig. 4C) presented a large overlap between cell types, of which several terms were associated with immune system responses (such as infections and diseases). Among these terms, we highlight FcγR-mediated phagocytosis, a signalling pathway that interplays adaptive and innate immune responses mediated by antibodies. We also noticed that, within this pathway, lymphocytes presented only upregulated proteins, while monocytes were more diverse in their response (Fig. 4D). Only the monocyte infectome was enriched for COVID-19 disease, suggesting that these myeloid cells may play a pivotal role in the canonically activated pathways during the course of SARS-CoV-2 infection.

### SARS-CoV-2 infection modulates visceral and subcutaneous adipocyte proteomes

**Figure 5:**
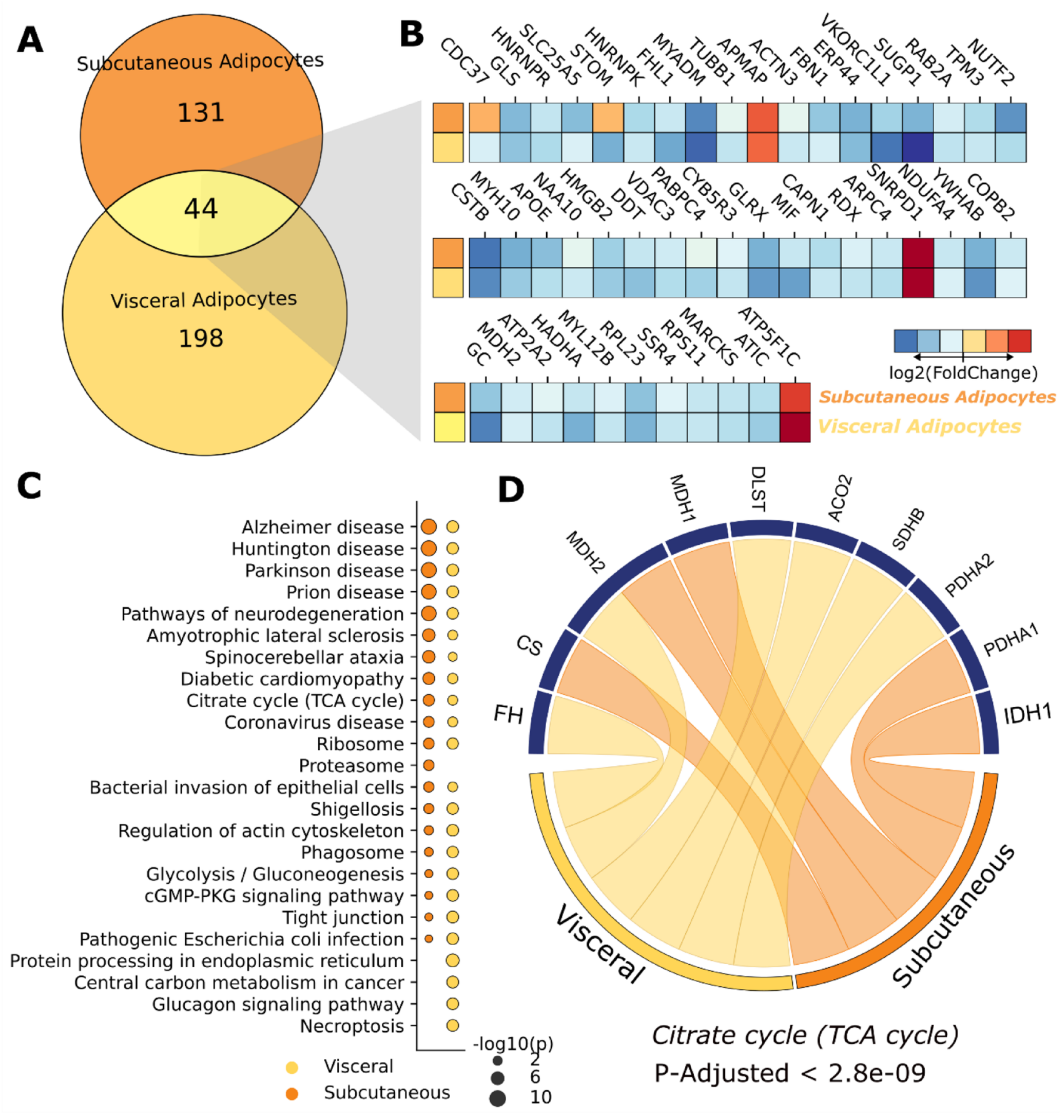
SARS-CoV-2 induces changes in the proteome of adipocytes. (**A**) Venn diagram showing differentially expressed proteins in visceral and subcutaneous adipocytes. (**B**) Heatmap of fold changes for proteins found in both adipocyte types. (**C**) Pair-wise comparison of top pathways enriched against KEGG database. Bubble size indicates adjusted *p*-value on a − log10 scale. (**D**) Upregulated (red) and downregulated (blue) proteins involved in the citrate (TCA) cycle.

Since visceral and subcutaneous adipocytes are known targets for SARS-CoV-2 infection and storage^33,34^ we investigated the proteomic modulations that occur in these cell types. 242 (visceral) and 175 (subcutaneous) proteins were found to be deregulated after SARS-CoV-2 infection. While nearly 60% of identified proteins were observed in both cell types, 44 proteins were dysregulated in both (Fig. 5A), most of which were in the same direction. No pathways were found to be enriched, despite the presence of many glucose metabolism proteins (Fig. 5B).

When comparing the top enriched pathways from both adipocyte types, only proteasome processes were unique to visceral adipocytes; subcutaneous adipocytes presented more unique processes, namely protein processing in the endoplasmic reticulum, central carbon metabolism in cancer, glucagon signalling pathway, and necroptosis (Fig. 5C). Despite these differences, TCA cycle proteins were found downregulated in both types, indicating dysregulated NAD^+^ and FAD reduction, culminating in lowered ATP production (Fig. 5D).

## Discussion

COVID-19 was initially classified as a respiratory disease; however, many studies have since proven that SARS-CoV-2 has the capacity to infect several other cell types and tissues. It has since been classified as a systemic disease with effects including kidney failure, permanent liver damage, diarrhoea, and neurological symptoms and sequelae. An increasing necessity to better understand the molecular and biochemical mechanisms triggered by SARS-CoV-2 infection throughout the human body led us to create the SARS-CoV-2 Infectome Atlas, in which several human cell types infected by SARS-CoV-2 were analysed.

SARS-CoV-2 has been found in human brain tissue collected *postmortem* and has also been shown to infect CNS models such as organoids and hamster brains^11,35^. Nonetheless, there is still no consensus as to if the brain alterations are directly related to the viral infection or if it is a secondary effect. Moreover, the mechanisms utilised by the virus to infect these cells and which changes are triggered or provoked by its replication are still a matter of discussion. This work reinforces the ability of SARS-CoV-2 to infect different CNS cell types and subsequently trigger proteomic changes. NPC-derived neurons exhibited the most affected model by the size of its infectome. Around 25% of the total identified proteomes from NSC-derived astrocytes, neurons, and differentiated SH-SY5Y neuroblastoma cells were deregulated after infection. Neurons or astrocytes have been hypothesised to be some of the first affected cells within the CNS due to their proximity to the blood–brain barrier (BBB), which would act as the port of entry of the virus into the CNS. Another noncompeting hypothesis is that axonal projections that reach the olfactory bulb may carry the virus into the CNS, thereby bypassing the BBB^5,36^.

Furthermore, infection of the CNS increases IL-6 levels, primarily as a response by astrocytes and microglia, potentially reducing the integrity of the BBB and promoting further viral invasion into the CNS^37,38^. This increase in IL-6 levels has been observed in the cerebrospinal fluid of patients with COVID-19^39,40^. In this study, the astrocyte infectome enriched for biological pathways involved in other chronic inflammatory diseases and responses to infectious agents, suggesting that these cells may be involved in the primary response to SARS-CoV-2 infection.

Within brain tissue, SARS-CoV-2 presence is concentrated in astrocytes despite a more accentuated proteomic change in neurons^11^. While comparing results from the enrichment analysis, NPC-derived neurons and differentiated SH-5YSY cells had a higher overlap of enriched pathways than with astrocytes, likely due to ontological similarity. Many of the enriched pathways were associated with neurodegenerative disease, glucose energy metabolism, COVID-19 disease, and ribosomal translational functions. The enrichment for neurodegenerative diseases could potentially be explained by increases in cell death and apoptosis.

Energy metabolism was affected in all CNS cells analysed in this study, making it a pathway of interest for further study. Proteins belonging to glycolytic pathways were generally upregulated in neurons and SH-5YSY cells, while in astrocytes they were downregulated. Calmodulin-dependent protein kinase (CAMK2D) was found upregulated in astrocytes, a protein that plays an important role in glutamatergic synapses through the post NMDA receptor activation events, possibly representing a higher uptake of glutamate from the extracellular medium.

In all cell types, PHGDH was found upregulated (Fig. 2B), a protein which catalyses the synthesis of L-serine, the precursor to D-serine, which is essential for NMDA receptor function throughout neurodevelopment. The upregulation of PHGDH in these cells could be related to an increased consumption of glutamate by the infected CNS cells to offset the increased energy demands resulting from the replication of viral particles. Crunfli et al. showed an imbalance in the glutamate/glutamine interplay between SARS-CoV-2 infected astrocytes and neurons^11^ and proposed that the higher mortality observed in neurons and SH-5YSY cells after being exposed to medium conditioned with infected astrocytes is due to a disruption of this feedback system between astrocytes and neurons.

More than half of COVID-19 patients report gastrointestinal symptoms, most commonly diarrhoea, but also including anorexia, nausea, vomiting, abdominal pain and gastrointestinal bleeding^41^. ACE2 is abundantly expressed in the intestinal epithelial cell cytoplasm^42^, enabling SARS-CoV-2 to directly target gastrointestinal cells, specifically gastric and intestinal epithelial cells^43^. Based on this evidence and the various gastroenterological symptoms, we chose to investigate the cellular response to infection in epithelial intestinal cells (CACO-2) and hepatocytes when creating our SARS-CoV-2 infection proteome map. The pathway analysis from the 332 proteins in the CACO-2 infectome mainly enriched for pathways related to chronic inflammatory diseases, energy metabolism, and biosynthesis of amino acids, highlighting the necessity of SARS-CoV-2 to induce changes in energy and protein metabolism to quickly replicate and create new viral particles.

Bojkova et al. used the same cell line labelled with stable isotopic amino acids (SILAC) to study the proteomic profile after SARS-CoV-2 infection^44^, discovering 459 proteins dysregulated by viral infection, enriching for glycolysis and nucleotide metabolism pathways. They also tested inhibitors of protein translation, RNA splicing, glycolysis, and nucleotide synthesis at non-toxic concentrations, which, in line with our findings, inhibited viral replication. Another study raised the hypothesis that, upon the ACE2-mediated entry of SARS-CoV-2 into the gastrointestinal tract via the small intestine, viral invasion and expansion occur, triggering gastrointestinal inflammation and potentially evolving into a strong cytokine response^45^.

Within the gastroenterological system, the liver is the organ that is considered to be most impacted by COVID-19, with approximately 58–75% of patients presenting hepatic injury and/or increased levels of transaminases^46–49^. While patients with COVID-19 generally present only moderate liver damage, more severe cases have been reported^47,48^. Wang and colleagues were the first to provide evidence of cytopathy in hepatocytes caused by SARS-CoV-2 infection that could cause liver impairment in the infected host^49^. Our analysis indeed shows that SARS-CoV-2 elicits proteomic changes in the HepG2 cell line, affecting proteins related to energy metabolism, the proteasome, amino acid biosynthesis, and pyruvate metabolism. When filtering for proteins associated with carbon metabolism in hepatocytes, there was an increase in proteins involved in pyruvate production, indicating once again that glucose metabolism is crucial during viral replication.

Adipose tissue has also been associated with SARS-CoV-2 infection and the subsequent inflammatory response^34,50–52^. Since the entry of SARS-CoV-2 into the digestive system, obesity, and high quantities of adipose tissue are risk factors and indicators of poor prognosis for severe COVID-19 and its evolution^53^, we evaluated infectomes of primary stromal-vascular cells isolated from subcutaneous (abdomen) or visceral (omentum) adipose tissues. Subcutaneous and visceral adipose tissue cells presented different responses to infection, with only 44 proteins in common. A pathway enrichment analysis highlighted changes in pyruvate metabolism, carbon metabolism, glycolysis/gluconeogenesis, and the TCA cycle in both cell types.

These dysregulations could be associated with the hyperglycemia that is often reported in acute COVID-19 patients and other metabolic dysfunctions that is seen in this disease^54^. Hospitalised COVID-19 patients with hyperglycemia also presented a higher prevalence of insulin resistance when compared to ARDS (acute respiratory distress syndrome)-positive and ARDS-negative controls, due to changes in adipokine levels (adiponectin and leptin), instead of beta-cell function^54^. This was also observed in other studies that analysed adipokine levels in infected hamsters^55,56^. The energy metabolism dysfunctions seen in both adipocyte infectomes could be disrupting the proper functioning of these cells, inducing changes in adipokine secretion. When epithelial intestinal cells (CACO-2), hepatocytes (HepG2), and both subcutaneous and visceral adipose tissue were compared, only MDH2 (malate dehydrogenase 2) was found in all infectomes, being upregulated in CACO-2 cells and downregulated in all other cell types (Supplementary Fig. 8).

The immune response that is mounted upon SARS-CoV-2 infection is against the spike and nucleocapsid viral components and seeks to eliminate the virus from the host^57^. Lower levels of neutralising antibodies in circulation and a progression to severe COVID-19 are strongly associated with an exacerbated inflammatory response, alterations in the immune system response characterised by protein synthesis reduction, a “cytokine storm”, lymphocytopenia, and T-cell exhaustion^58,59^. Immune responses occur through two different mechanisms: the innate immune response, mediated by macrophages, dendritic cells, and natural killer cells, and/or the adaptive immune response, mediated by T-cells and B-cells.

To cover as many of the proteomic changes brought about by SARS-CoV-2 infection as possible, we used human primary T-cells and monocytes as infection models, thereby screening both innate and adaptive responses^8,12,28^. The resulting pathway analyses showed a high similarity between the T-cells and monocytes, enriching for several pathways related to response to infection, phagocytosis, neutrophil cellular trap formation, and, once again, energy metabolism. Ribosomal changes and coronavirus disease were identified only in monocytes, likely since monocytes are responsible for the activation of macrophages and produce the initial immune response. In agreement with this hypothesis, chemokine signalling activation and leukocyte transendothelial migration were both enriched exclusively in monocytes. The T-cell infectome also exclusively enriched for certain biological pathways, which included apoptotic and necroptotic pathways, supporting a study that saw a decrease in CD4^+^ T-cell viability after SARS-CoV-2 infection^12^. As SARS-CoV-2 replication is dependent on the presence of glucose, this highlights the many pathways involved in glucose metabolism that were enriched in both cell types. Moreover, this suggests that energy production dysfunctions could be directly related to an exacerbated immune response and resulting cytokine storm^28^.

When all cell types were compared together, a large overlap was observed in identified proteins; however, when only dysregulated proteins were considered, this overlap vanished (Supplementary Fig. 5). Nie et al. observed a similar trend in their proteomic analysis of seven tissue types from patients who died from COVID-19^8^. They showed that only 39 proteins were commonly dysregulated of a total of 11,394 identified proteins, representing less than 0·004% of identifications. Despite the lack of overlap of individual proteins, energy metabolism was affected in every infectome (Supplementary Fig. 9), which strongly supports the hypothesis that glucose metabolism is a key factor for SARS-CoV-2 replication inside the host cell. This hypothesis is further supported by several studies that have reported disturbances in energy production in tissues and cells infected by SARS-CoV-2^8,11,28^.

To facilitate the future investigation of all the data collected and analysed in this work, we have constructed an open-source tool to facilitate the search for proteins found to be dysregulated in each cell type and tissue type. Other data include their relative abundance in comparison to controls and the biological pathways they enrich, all of which is freely available at https://reisdeoliveira.shinyapps.io/Infectome_App/. Future proteomic analyses can then be submitted to this database to expand this SARS-CoV-2 Infectome Atlas.

## Methods

### Induced pluripotent stem cells (IPSCs) purchase

Neural Stem Cells were purchased from Cellossaurus (hES, BR-1 cell line, RRID:CVCL_C062).

### Generation of human astrocytes (hES-derived)

Differentiation of glial progenitor cells was performed from neural stem cells (NSC) derived from pluripotent human embryonic stem cells (hES, BR-1 cell line, RRID:CVCL_C062)^14^, according to the method published by Trindade et al*.*^15^ NSCs were cultured on plates coated with Geltrex Matrix (Thermo Fisher Scientific, MA, USA) using 1:1 Neurobasal™: advanced DMEM/F12 medium and 2% neural induction supplement. Upon reaching 50% confluence, the medium was changed to DMEM/F12 (Dulbecco’s modified Eagle medium/F12), 1% N2 supplement, 1% foetal bovine serum (FBS), and 1% penicillin–streptomycin and cells were maintained at 37 °C in humidified air with 5% atmospheric CO_2_ for 21 days. At this stage, cells were considered glial progenitor cells (GPC).

Subsequently, GPCs were plated at low density (30–40% confluence) on Geltrex-coated plates with DMEM/F12 medium, 1% GlutaMAX Supplement, 10% FBS and 1% penicillin–streptomycin. The differentiation medium was replaced every 2–3 days. After 4 weeks of differentiation, the cells were considered mature astrocytes. These cells were plated on Geltrex-coated coverslips at a density of 4 × 10^4^ cells for immunostaining assays (24-well plates) or 2·5 × 10^5^ cells for viral load, proteomic, and metabolomic analysis (6-well plates).

All products used for cell culture are from Thermo Fisher Scientific, MA, USA. The characterization of the BR-1 lineage as astrocyte cells has been previously described elsewhere^14–17^. We generated eight batches of human astrocytes from BR-1-derived NSCs. The NSCs were of different passages and were used as biological replicates in independent experiments. Our internal control showed that approximately 97% of the neural stem cell-derived astrocytes in culture expressed GFAP, 80·4% expressed vimentin, and 12·9% expressed SOX-2 (markers of progenitor cells). The neural stem cell-derived astrocyte culture expressed more astrocytic markers than progenitor cell markers, indicating an excellent effectiveness of human astrocyte generation. Method described in our previous work^11^.

### Differentiation of the SH-SY5Y human neuroblastoma cell line

The SH-SY5Y cell line (SH-SY5Y ATCC-CRL-2266, RRID:CVCL_0019), kindly donated by Prof. Dr. Gustavo J. S. Pereira (Federal University of São Paulo, UNIFESP), was cultivated using a previously documented neuronal differentiation protocol^18–20^ using DMEM/F12 medium, 10% FBS and 1% penicillin–streptomycin at 37 °C in humidified air with 5% atmospheric CO_2_. The SH-SY5Y cells were plated and, upon reaching 25–30% confluency, the medium was changed to neuronal differentiation medium consisting of DMEM/F12 with 1% FBS and 10 µM retinoic acid (Sigma Aldrich). The differentiation medium was replaced every 2–3 days over the course of 2 weeks. These differentiated SH-SY5Y cells are known to more closely relate to adrenergic neurons, but they also express dopaminergic markers^18^.

### NSC differentiation into neurons

Human NSC-derived neurons were cultivated following the protocol described by Thermo Fisher Scientific^21–23^. NSCs were plated on Geltrex-coated plates and maintained with NEM medium at 37 °C in humidified air with 5% atmospheric CO_2_. Upon reaching 40% confluency, the medium was changed to neuronal differentiation medium consisting of 1:1 DMEM/F12: neurobasal medium with 1% B27 supplement (Thermo Fisher Scientific, Carlsbad, CA, USA) and 1% GlutaMAX (Thermo Fisher Scientific, Carlsbad, CA, USA). The medium was renewed every four days over the course of 20 days by removing half of the volume and replacing the same volume of fresh medium. Medium renewal was performed in this manner since factors secreted by the differentiating cells are important for successful differentiation. Two control cell lines were used: GM23279A, obtained from a female subject (available at Coriell; RRID:CVCL_F178) and BR-1 (RRID:CVCL_C062)^14^. Both cell lines were cultivated following previously published protocols^16,24–26^ and have been extensively characterised elsewhere^21–23^. We also used FACS to analyse cellular markers and found a *bona fide* neuronal phenotype with the expression of the neuronal markers synaptophysin (75·9% of cells), MAP2 (99·9%), and β-tubulin (99%), as well as astrocytic marker GFAP (8·1%).

### Human adipose tissue mesenchymal stem cell isolation, culture and adipocyte differentiation

Patients, samples collection and adipocyte differentiation were previous described in our work ^13^. For this work it was used the same samples but with independent runs in the LC-MS/MS system. Briefly, human adipose tissue-derived mesenchymal stem cells (hADSCs) were isolated from abdominal subcutaneous adipose tissue and visceral omental adipose tissue of three individuals who underwent abdominal surgery (i.e., bariatric surgery or cholecystectomy) at the Clinical Hospital of the University of Campinas, Campinas, SP, Brazil. These cells were isolated prior to the COVID-19 pandemic and hence the donors were not infected with SARS-CoV-2. The subjects received written and oral information before providing written informed consent for the collection of the biopsy and use of the tissues. All material used in the procedure was sterile. Biopsies were collected during surgery and transported to the laboratory within 30 min in sealed, sterile falcon tubes for initiation of the procedure of stromal-vascular fraction isolation. The tissue was weighed, cut into small pieces on a petri dish, and digested with 25–30 mL of lysis buffer (1 mg/mL collagenase in HBSS containing 2% BSA, filtered through a 0·22 μm filter) at 37 °C for 30–50 min with slight agitation, until homogeneous. The homogenate was filtered through a 250 μm filter and collected in a sterile falcon tube. After a brief rest period (approximately 5 min), the infranatant containing the stromal-vascular fraction was collected using a Pasteur pipette and centrifuged for 5 min at 200×*g* and 4 °C. The supernatant was discarded, and the cell pellet was washed with HBSS. The centrifugation and washing steps were repeated twice. The pellet was then resuspended in BM-1 medium (ZenBio) with 10% foetal bovine serum (FBS) and 1% penicillin/streptomycin (penicillin 10,000 units/mL, streptomycin 10 mg/mL).

Cells were cultured at 37 °C and 5% atmospheric CO_2_ until semi-confluent (80–90%). They were then trypsinized and frozen in freezing medium (10% DMSO, 50% FBS, and 40% basal culture medium) and stored in a liquid nitrogen biorepository for further assays (e.g., adipocyte differentiation followed by exposure with agents, lipid quantification, determination of cell viability, gene expression analysis, proteomics, immunodetection of proteins, quantification of cell secretion products, and metabolic analysis).

hADSCs were thawed in BM-1 medium supplemented with 10% FBS, 1% penicillin/streptomycin, 17 ng/mL bFGF, and 15 ng/mL BMP4, and cultured at 37 °C and 5% atmospheric CO_2_. Cells were expanded (i.e., split 1–3 times) and then seeded onto 24-well plates at a density of 40,000 cells/cm^2^for adipocyte differentiation. When cells reached 100% confluency (day 0), the medium was replaced with BM-1 supplemented with 3% FBS, 1% penicillin/streptomycin, 0·1 µM dexamethasone, 500 µM 3-isobutyl-1 methylxanthine (IBMX), 20 nM insulin, 5 nM triiodothyronine (T3) and 10 ng/mL BMP4. Cells were cultured for 7 days, and the medium was changed every 2–3 days. On day 7, the medium was replaced with BM-1 supplemented with 3% FBS, 1% penicillin/streptomycin, 0·1 µM dexamethasone, 20 nM insulin, 5 nM T3 and 10 ng/mL BMP4 until the cells were fully differentiated (day 10). Cells were then cultured for an additional 3 days with BM-1 supplemented with 3% FBS and 1% penicillin/streptomycin before being subjected to the assays described below.

### Virus strain

The HIAE-02-SARS-CoV-2/SP02/human/2020/BRA (GenBank accession number MT126808·1) virus strain was used for all in vitro experiments. The virus was isolated from the first confirmed case of COVID-19 in Brazil and kindly donated by Prof. Dr. Edison Durigon (ICB-USP). VSV-eGFP-SARS-CoV-2 was engineered and donated by Prof. Dr. Sean P.J. Whelan (Department of Medicine, Washington University School of Medicine, St. Louis, MO, USA) for SARS-CoV-2 entry experiments^27^. Viral stock was propagated in Vero CCL-81 cells (ATCC, RRID:CVCL_0059), cultivated in DMEM supplemented with 10% heat-inactivated FBS and 1% penicillin and streptomycin (Gibco, Walthmam, MA, USA), and incubated at 37 °C with 5% atmospheric CO_2_. Viral titer was determined by the plaque-forming assay using Vero cells.

### Human lymphocyte isolation and mixed lymphocyte reaction

PBMCs isolated from buffy coats from healthy volunteers were incubated at 37 °C and 5% atmospheric CO_2_ for 2 h to allow monocyte adherence to the plate surface. Non-adherent cells in suspension were collected and stained with carboxyfluorescein succinimidyl ester (Cell-Trace CFSE). Cells were incubated with CFSE diluted in pre-warmed PBS to the desired concentrations at 37 °C for 15 min, resuspended in fresh, pre-warmed medium for 30 min, and then washed with PBS, according to the manufacturer’s recommendations. Is this work it was reanalalyzed the proteomic data obtained by our previous work. Patients steatments and ethical issues are also described at Codo et al.^28^.

### Human colon cancer cell line (CACO-2) culture

Human colon cancer cell line CACO-2 (RRID:CVCL_0025) was obtained from the American Type Culture Collection (ATCC). Cells were maintained in Dulbecco’s modified Eagle medium (Gibco) supplemented with 20% foetal bovine serum (FBS) and 1% penicillin–streptomycin at 37 °C and 5% atmospheric CO_2_. CACO-2 cells were exposed to SARS-CoV-2 for 1 h at an MOI of 0·1 (P3 21/05/20—10^4^ UFF/mL) under gentle agitation at room temperature. After viral adsorption, cells were washed twice with phosphate-buffered saline (PBS) and incubated with DMEM supplemented with 20% FBS and 1% penicillin and streptomycin for 24 h at 37 °C and 5% atmospheric CO_2_. This method section was published on Crunfli et al. ().

### Human liver carcinoma cell line (HepG2) culture

HepG2 cells (human liver carcinoma cell line HepG2/CD81++) were cultured for 3 days in Dulbecco’s modified Eagle medium (DMEM; Vitrocell, Campinas, SP, Brazil), enriched with 10% (v/v) foetal bovine serum, under a humidified conditions with 5% atmospheric CO_2_ at 37 °C. After reaching 80% confluence, the cells underwent viral infection of 0·1 MOI, prior to preparation for proteomic analyses, as described below.

### Blood sample collection and lymphocyte separation

Each COVID-19 patient had heparin and plain blood tubes collected. Whole blood, serum, and plasma samples were separated. Peripheral blood mononuclear cells (PBMCs) from patients and buffy coats were obtained via Histopaque-1077 density gradient (Sigma-Aldrich). Samples were diluted in Hanks balanced salt solution (1:1) and gently poured into 15- or 50-mL conical tubes containing 3 or 10 mL of Histopaque, respectively. Samples were centrifuged at 1400 rpm for 30 min at 4 °C without acceleration or braking. After the PBMC layer was transferred to new tube, lymphocytes were sorted and incubated overnight with RPMI 1640 (Gibco) containing 10% foetal bovine serum (FBS) and 1% penicillin–streptomycin (P/S), at 37 °C with 5% atmospheric CO_2_. Is this work it was reanalalyzed the proteomic data obtained by our previous work. Patients steatments and ethical issues are also described at Davanzo et al.^12^.

### In vitro astrocyte infection

Astrocytes were infected with SARS-CoV-2 for 1 h (MOI of 0·1 for proteomic, viral load, and bioenergetic assays) under gentle agitation at room temperature. After viral adsorption, cells were washed twice with phosphate-buffered saline (PBS) and incubated with DMEM/F12 supplemented with 10% FBS, 1% GlutaMAX, and 1% penicillin and streptomycin for 24 h at 37ºC and 5% atmospheric CO_2_.

### LC-MS/MS sample preparation, analyses, and data processing

All cellular experiments underwent the same preparation process, as follows. Cells were lysed chemically with lysis buffer (100 mM Tris–HCl, 1 mM EDTA, 150 mM NaCl, 1% Triton-X, and protease and phosphatase inhibitors) then mechanically with an ultrasonication probe during three cycles of 20 s with 90% frequency, on ice. The total protein extract was quantified by Pierce BCA protein assay (Thermo Fisher Scientific, MA, USA) according to the manufacturer’s instructions. 30 µg of total protein extract from each sample was transferred to a Microcon-10 centrifugal filter with a 10 kDa cutoff for FASP protein digestion^29^. In brief, proteins were reduced (10 mM dithiothreitol) at 56 °C for 40 min, alkylated (50 mM iodoacetamide) at room temperature for 20 min in the dark, and digested overnight with trypsin at 37 °C in 50 mM ammonium bicarbonate (AmBic), pH 8·0. Peptides were recovered from the filter in 50 mM AmBic and trypsin activity was quenched by adding formic acid (FA) to a final concentration of 1% (v/v), whereupon the peptides were desiccated in a SpeedVac vacuum concentrator and stored at -80 °C until analysis.

Digested peptides were resuspended in 0·1% FA. LC-MS/MS analyses were performed in an ACQUITY UPLC M-Class System (Waters Corporation, Milford, MA) coupled online to a Synapt G2-Si mass spectrometer (Waters Corporation, Milford, MA). 1 μg of peptides were loaded onto a trapping column (Symmetry C18 5 μm, 180 μm × 20 mm, Waters Corporation, Milford, MA) and subsequently separated in the analytical column (HSS T3 C18 1·8 μm, 75 μm × 150 mm; Waters Corporation, Milford, MA). For gradient elutions, 0·1% FA was used as eluent A and acetonitrile-FA (99·9% ACN:0·1% FA) as eluent B. A reversed phase gradient was carried out over a 120-min method, with a linear gradient running from 3 to 60% eluent B over 90 min at 300 nL/min. In the Synapt G2-Si, the peptide spectra were acquired by ion mobility-enhanced data-independent acquisition (HDMS^E^). Mass spectrometry analysis was performed in Resolution Mode, switching between low (4 eV) and high (25–60 eV) collision energies, using a scan time of 1·0 s per function over 50–2000 m/z. The wave velocity for ion mobility separation was 1000 m/s and the transfer wave velocity was 175 m/s. A human [Glu1]-Fibrinopeptide B standard (Waters Corporation, Milford, MA) was used as the reference lock-mass compound. Each sample was acquired in at least duplicate.

The raw data from each experiment were processed in Progenesis QI for Proteomics, version 4·0·× (Waters Corporation, Milford, MA). Tandem mass spectra were searched against a reviewed *Homo sapiens* proteome (UNIPROT, release 2020-04), using tolerance parameters of 20 ppm for precursor ions and 10 ppm for product ions. For peptide identification, carbamidomethylation of cysteine was set as a fixed modification and oxidation of methionine as a variable modification, up to two missed cleavages were permitted and the false discovery rate (FDR) was limited to 1%. Protein identification was performed using a minimum of one fragment ion per peptide, three fragment ions per protein, and one peptide per protein. Sample acquisition until this point was performed by experimenter blind to group assignment and outcome assessment. Analyses after this point were performed with predefined algorithms and cutoffs, reducing the possibility of experimenter-induced bias.

The label-free quantitative analysis was carried out using the relative abundance intensity of all peptides of a protein with at least one unique peptide after normalisation by all peptide intensities. The expression analysis was performed considering the technical replicates for each experimental condition, following the hypothesis that each group is independent. Proteins with ANOVA (p) ≤ 0·05 between groups were considered differentially expressed. Data integration and visualization was carried out in OmicScope package^62^ (v.1.2.2), using Progenesis Method and default parameters. The method applied for proteomics propose are better described at Crunfli et al.^11^.

### RNA extraction and viral load

Total RNA extraction was performed using TRI Reagent (Sigma, St Louis, USA) according to the manufacturer’s instructions. RNA concentration was determined by a DeNovix spectrophotometer and RNA integrity was assessed by visualisation of 28S and 18S ribosomal RNA on a 1% agarose gel. Reverse transcription was performed with 0·5 µg of RNA using a GoScript reverse transcriptase kit (Promega, Madison, WI, USA) according to the manufacturer’s instructions. qPCR was performed using astrocyte cDNA diluted 1:10 and the qPCR SybrGreen Supermix (Qiagen, Valencia, CA, USA) containing forward and reverse primers in RNAse-free water. All reactions were performed in a CFX384 Touch real-time PCR detection system (Biorad, Hercules, CA, USA) and cycling conditions were set as follows: 50 °C for 2 min; 95 °C for 10 min; (95 °C for 15 s; 60 °C for 1 min) × 40 cycles. To evaluate primer specificity, a melting curve analysis was performed by heating samples from 65 to 99 °C (1 °C increment changes at 5 s intervals). All sample measurements were performed in duplicate. Primers were designed with PrimerBlast and used at a concentration of 200 nM. Data were normalised to the expression of 18S (Fwd 5′ CCCAACTTCTTAGAGGGACAAG 3′; Rev 5′ CATCTAAGGGCATCACAGACC 3′) and the relative quantification value of each target gene was determined using a comparative CT method^30^. For virus detection, SARS-CoV-2 nucleocapsid N1 primers (Fwd 5′ CAATGCTGCAATCGTGCTAC 3′; Rev 5′ GTTGCGACTACGTGATGAGG 3′) were used as previously described^28,31^. Data were expressed as mean ± SEM. Statistical significance was calculated by a two-tailed unpaired Student’s t-test. All analyses were performed using GraphPad Prism 8·0 (San Diego, CA, USA) and a significance level of *p* ≤ 0·05 was adopted.

### Ethical approval

The protocols for human sample collection, storage, and analysis were conducted according to the guidelines of the Declaration of Helsinki and approved by the Institutional Research Ethics Committees of the University of São Paulo and University of Campinas (CAAE 48,836,721.3.0000.5440 and CAAE 78,577,417.8.0000.5404). The latter was confirmed by the National Ethics and Research Council. Procedures for sample collection, processing, and subject information are described below. Donors were not compensated.

### Supplementary Information

Supplementary Information 1.

Supplementary Table 1.

## Data Availability

The mass spectrometry proteomic data have been deposited to the ProteomeXchange Consortium via the PRIDE partner repository^60,61^ with dataset identifiers PXD023781, PXD030910, PXD0269521, PXD020967 and PXD050226. The unique primary cells and biopsies used in this study can only be shared upon approval by the local ethics committee and donor consent.

## References

[1] Geneva: World Health Organization. WHO COVID-19 Dashboard. WHO Health Emergency Dashboard. 2020. https://covid19.who.int/ Accessed 21 Dec 2022

[2] Zou X, Chen K, Zou J, Han P, Hao J, Han Z (2020). Single-cell RNA-seq data analysis on the receptor ACE2 expression reveals the potential risk of different human organs vulnerable to 2019-nCoV infection. Front Med..

[3] Varatharaj A, Thomas N, Ellul MA (2020). Neurological and neuropsychiatric complications of COVID-19 in 153 patients: A UK-wide surveillance study. Lancet Psychiatry.

[4] Lin L, Jiang X, Zhang Z (2020). Gastrointestinal symptoms of 95 cases with SARS-CoV-2 infection. Gut.

[5] Yachou Y, El Idrissi A, Belapasov V, Ait BS (2020). Neuroinvasion, neurotropic, and neuroinflammatory events of SARS-CoV-2: Understanding the neurological manifestations in COVID-19 patients. Neurol. Sci..

[6] Gupta A, Madhavan MV, Sehgal K (2020). Extrapulmonary manifestations of COVID-19. Nat. Med..

[7] Yang L, Liu S, Liu J (2020). COVID-19: Immunopathogenesis and Immunotherapeutics. Signal Transduct. Target Ther..

[8] Nie X, Qian L, Sun R (2021). Multi-organ proteomic landscape of COVID-19 autopsies. Cell.

[9] Kalejaiye TD, Bhattacharya R, Burt MA (2022). SARS-CoV-2 employ BSG/CD147 and ACE2 receptors to directly infect human induced pluripotent stem cell-derived kidney podocytes. Front. Cell Dev. Biol..

[10] Hoffmann M, Kleine-Weber H, Schroeder S (2020). SARS-CoV-2 cell entry depends on ACE2 and TMPRSS2 and is blocked by a clinically proven protease inhibitor. Cell.

[11] Crunfli F, Carregari VC, Veras FP (2022). Morphological, cellular, and molecular basis of brain infection in COVID-19 patients. Proc. Natl. Acad. Sci. U. S. A..

[12] Davanzo, G. G., Codo, A. C. & Brunetti, N. S., et al. SARS-CoV-2 uses CD4 to infect T helper lymphocytes. (2020). 2020.09.25.20200329.

[13] Saccon TD, Mousovich-Neto F, Ludwig RG (2022). SARS-CoV-2 infects adipose tissue in a fat depot- and viral lineage-dependent manner. Nat. Commun..

[14] Fraga AM, Sukoyan M, Rajan P (2011). Establishment of a Brazilian line of human embryonic stem cells in defined medium: Implications for cell therapy in an ethnically diverse population. Cell Transplant..

[15] Trindade P, Loiola EC, Gasparotto J (2020). Short and long TNF-alpha exposure recapitulates canonical astrogliosis events in human-induced pluripotent stem cells-derived astrocytes. Glia.

[16] Ledur PF, Karmirian K, da Pedrosa C (2020). Zika virus infection leads to mitochondrial failure, oxidative stress and DNA damage in human iPSC-derived astrocytes. Sci. Rep..

[17] Yan Y, Shin S, Jha BS (2013). Efficient and rapid derivation of primitive neural stem cells and generation of brain subtype neurons from human pluripotent stem cells. Stem Cells Transl. Med..

[18] Kovalevich J, Langford D (2013). Considerations for the use of SH-SY5Y neuroblastoma cells in neurobiology. Methods Mol. Biol..

[19] Shipley MM, Mangold CA, Szpara ML (2016). Differentiation of the SH-SY5Y human neuroblastoma cell line. J. Vis. Exp..

[20] Xicoy H, Wieringa B, Martens GJM (2017). The SH-SY5Y cell line in Parkinson’s disease research: A systematic review. Mol. Neurodegener.

[21] Brewer GJ (1995). Serum-free B27/neurobasal medium supports differentiated growth of neurons from the striatum, substantia nigra, septum, cerebral cortex, cerebellum, and dentate gyrus. J. Neurosci. Res..

[22] Elkabetz Y, Studer L (2008). Human ESC-derived neural rosettes and neural stem cell progression. Cold Spring Harb. Symp. Quant. Biol..

[23] Trujillo CA, Schwindt TT, Martins AH, Alves JM, Mello LE, Ulrich H (2009). Novel perspectives of neural stem cell differentiation: From neurotransmitters to therapeutics. Cytometry A.

[24] Casas BS, Vitória G, do Costa MN (2018). hiPSC-derived neural stem cells from patients with schizophrenia induce an impaired angiogenesis. Transl. Psychiatry.

[25] Goto-Silva L, Ayad NME, Herzog IL (2019). Computational fluid dynamic analysis of physical forces playing a role in brain organoid cultures in two different multiplex platforms. BMC Dev. Biol..

[26] White JA, Krzystek TJ, Hoffmar-Glennon H (2020). Excess Rab4 rescues synaptic and behavioral dysfunction caused by defective HTT-Rab4 axonal transport in Huntington’s disease. Acta Neuropathol. Commun..

[27] Case JB, Rothlauf PW, Chen RE (2020). Neutralizing antibody and soluble ACE2 inhibition of a replication-competent VSV-SARS-CoV-2 and a clinical isolate of SARS-CoV-2. Cell Host Microbe.

[28] Codo AC, Davanzo GG, de Monteiro LB (2020). Elevated glucose levels favor SARS-CoV-2 infection and monocyte response through a HIF-1α/glycolysis-dependent axis. Cell Metab..

[29] Distler U, Kuharev J, Navarro P, Tenzer S (2016). Label-free quantification in ion mobility-enhanced data-independent acquisition proteomics. Nat. Protoc..

[30] Schmittgen TD, Livak KJ (2008). Analyzing real-time PCR data by the comparative C(T) method. Nat. Protoc..

[31] Won J, Lee S, Park M (2020). Development of a laboratory-safe and low-cost detection protocol for SARS-CoV-2 of the coronavirus disease 2019 (COVID-19). Exp. Neurobiol..

[32] Kanehisa M, Goto S (2000). KEGG: Kyoto encyclopedia of genes and genomes. Nucleic Acids Res.

[33] Ryan PM, Caplice NM (2020). Is adipose tissue a reservoir for viral spread, immune activation, and cytokine amplification in coronavirus disease 2019?. Obesity (Silver Spring).

[34] Kruglikov IL, Scherer PE (2020). The role of adipocytes and adipocyte-like cells in the severity of COVID-19 infections. Obesity (Silver Spring).

[35] Jacob F, Pather SR, Huang W-K (2020). Human pluripotent stem cell-derived neural cells and brain organoids reveal SARS-CoV-2 neurotropism predominates in choroid plexus epithelium. Cell Stem Cell.

[36] Alquisiras-Burgos I, Peralta-Arrieta I, Alonso-Palomares LA, Zacapala-Gómez AE, Salmerón-Bárcenas EG, Aguilera P (2021). Neurological complications associated with the blood–brain barrier damage induced by the inflammatory response during SARS-CoV-2 infection. Mol. Neurobiol..

[37] Koyuncu OO, Hogue IB, Enquist LW (2013). Virus infections in the nervous system. Cell Host Microbe.

[38] Erta M, Quintana A, Hidalgo J (2012). Interleukin-6, a major cytokine in the central nervous system. Int. J. Biol. Sci..

[39] Rochfort KD, Collins LE, Murphy RP, Cummins PM (2014). Downregulation of blood–brain barrier phenotype by proinflammatory cytokines involves NADPH oxidase-dependent ROS generation: Consequences for interendothelial adherens and tight junctions. PLoS ONE.

[40] Zhang J, Sadowska GB, Chen X (2015). Anti-IL-6 neutralizing antibody modulates blood–brain barrier function in the ovine fetus. FASEB J.

[41] Lei H-Y, Ding Y-H, Nie K (2021). Potential effects of SARS-CoV-2 on the gastrointestinal tract and liver. Biomed. Pharmacother..

[42] Zhou L, Niu Z, Jiang X (2020). SARS-CoV-2 targets by the pscRNA profiling of ACE2, TMPRSS2 and furin proteases. iScience.

[43] Xiao F, Tang M, Zheng X, Liu Y, Li X, Shan H (2020). Evidence for gastrointestinal infection of SARS-CoV-2. Gastroenterology.

[44] Bojkova D, Klann K, Koch B (2020). Proteomics of SARS-CoV-2-infected host cells reveals therapy targets. Nature.

[45] Zhang H, Li H-B, Lyu J-R (2020). Specific ACE2 expression in small intestinal enterocytes may cause gastrointestinal symptoms and injury after 2019-nCoV infection. Int. J. Infect Dis..

[46] Huang C, Wang Y, Li X (2020). Clinical features of patients infected with 2019 novel coronavirus in Wuhan, China. Lancet.

[47] Chen N, Zhou M, Dong X (2020). Epidemiological and clinical characteristics of 99 cases of 2019 novel coronavirus pneumonia in Wuhan, China: A descriptive study. Lancet.

[48] Bangash MN, Patel J, Parekh D (2020). COVID-19 and the liver: Little cause for concern. Lancet Gastroenterol. Hepatol..

[49] Wang Y, Liu S, Liu H (2020). SARS-CoV-2 infection of the liver directly contributes to hepatic impairment in patients with COVID-19. J. Hepatol..

[50] Wilk AJ, Lee MJ, Wei B (2021). Multi-omic profiling reveals widespread dysregulation of innate immunity and hematopoiesis in COVID-19. J. Exp. Med..

[51] Zickler M, Stanelle-Bertram S, Ehret S (2022). Replication of SARS-CoV-2 in adipose tissue determines organ and systemic lipid metabolism in hamsters and humans. Cell Metab..

[52] Basolo A, Poma AM, Bonuccelli D (2022). Adipose tissue in COVID-19: Detection of SARS-CoV-2 in adipocytes and activation of the interferon-alpha response. J. Endocrinol. Invest..

[53] Stefan N, Birkenfeld AL, Schulze MB, Ludwig DS (2020). Obesity and impaired metabolic health in patients with COVID-19. Nat. Rev. Endocrinol..

[54] Reiterer M, Rajan M, Gómez-Banoy N (2021). Hyperglycemia in acute COVID-19 is characterized by insulin resistance and adipose tissue infectivity by SARS-CoV-2. Cell Metab..

[55] Chan JF-W, Zhang AJ, Yuan S (2020). Simulation of the clinical and pathological manifestations of coronavirus disease 2019 (COVID-19) in a golden Syrian hamster model: Implications for disease pathogenesis and transmissibility. Clin. Infect. Dis..

[56] Sia SF, Yan L-M, Chin AWH (2020). Pathogenesis and transmission of SARS-CoV-2 in golden hamsters. Nature.

[57] Zhang X, Tan Y, Ling Y (2020). Viral and host factors related to the clinical outcome of COVID-19. Nature.

[58] Arunachalam PS, Wimmers F, Mok CKP (2020). Systems biological assessment of immunity to mild versus severe COVID-19 infection in humans. Science.

[59] Robbiani DF, Gaebler C, Muecksch F (2020). Convergent antibody responses to SARS-CoV-2 in convalescent individuals. Nature.

[60] Perez-Riverol Y, Csordas A, Bai J (2019). The PRIDE database and related tools and resources in 2019: Improving support for quantification data. Nucleic Acids Res..

[61] Perez-Riverol Y, Bai J, Bandla C (2022). The PRIDE database resources in 2022: A hub for mass spectrometry-based proteomics evidences. Nucleic Acids Res..

[62] Reis-de-Oliveira, G., *et al*. OmicScope (v1.2.2). Zenodo (2023). 10.5281/zenodo.8074722

