# Supplementary material for: Diving into the proteomic atlas of SARS-CoV-2 infected cells
Source: Sci Rep. 2024 Mar 28;14:7375. doi: 10.1038/s41598-024-56328-3 (PMC10978884; doi:10.1038/s41598-024-56328-3)
Supplement: Supplementary file 1 — Supplementary Information 1. [file 41598_2024_56328_MOESM1_ESM.docx]

Diving into the proteomic atlas of SARS-CoV-2 infected cells

Victor C. Carregari1#, Guilherme Reis-de-Oliveira1#, Fernanda Crunfli1, Bradley J. Smith1, Gabriela Fabiano de Souza2, Stéfanie Primon Muraro2, Veronica M. Saia-Cereda1, Pedro H. Vendramini1, Paulo A. Baldasso1,3, Lícia C. Silva-Costa1, Giuliana S. Zuccoli1, Caroline Brandão-Teles1, André Antunes1, Aline F. Valença1, Gustavo G. Davanzo4, João Victor Virgillio-da-Silva5,6, Thiago dos Reis Araújo7, Raphael Campos Guimarães6,7, André R. L. Damásio3, Luiz Osório S. Leiria5,6, Marco Aurélio R. Vinolo7,8,9, Alessandro S. Farias10,11,12, Pedro M. Moraes-Vieira 4,7,12, Marcelo A. Mori3,5,9, José Luiz P. Módena2, Daniel Martins-de-Souza1,3,10,12*

1. Laboratory of Neuroproteomics, Department of Biochemistry and Tissue Biology, Institute of Biology, University of Campinas (UNICAMP), Campinas, São Paulo, Brazil
2. Laboratory of Emerging Viruses, Department of Genetics, Evolution, Microbiology and Immunology, Institute of Biology, University of Campinas (UNICAMP), São Paulo, Brazil
3. Department of Biochemistry and Tissue Biology, Institute of Biology, University of Campinas (UNICAMP), Campinas, São Paulo, Brazil
4. Laboratory of Immunometabolism, Department of Genetics, Evolution, Microbiology and Immunology, Institute of Biology, University of Campinas (UNICAMP), São Paulo, Brazil
5. Department of Pharmacology, Ribeirão Preto Medical School (FMRP), University of São Paulo (USP), Ribeirão Preto, São Paulo, Brazil
6. Center for Research in Inflammatory Diseases, Ribeirão Preto, SP, Brazil
7. Obesity and Comorbidities Research Center (OCRC), Campinas, São Paulo, Brazil
8. Laboratory of Immunoinflammation, Department of Genetics, Microbiology and Immunology, Institute of Biology, University of Campinas (UNICAMP), Campinas, São Paulo, Brazil
9. Instituto Nacional de Biomarcadores em Neuropsiquiatria (INBION), Conselho Nacional de Desenvolvimento Científico e Tecnológico, São Paulo 05403-000, Brazil
10. D’Or Institute for Research and Education (IDOR), São Paulo 04501-000, Brazil
11. Autoimmune Research Laboratory, Department of Genetics, Microbiology and Immunology, Institute of Biology, University of Campinas (UNICAMP), Campinas, São Paulo, Brazil
12. Experimental Medicine Research Cluster (EMRC), University of Campinas (UNICAMP), Campinas, São Paulo, Brazil
13. Laboratory of Immunometabolism, Department of Genetics, Evolution, Microbiology and Immunology, Institute of Biology, University of Campinas (UNICAMP), São Paulo, Brazil

Supplementary Material

- 1. **B)**


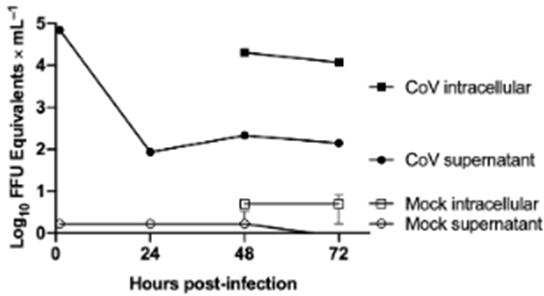

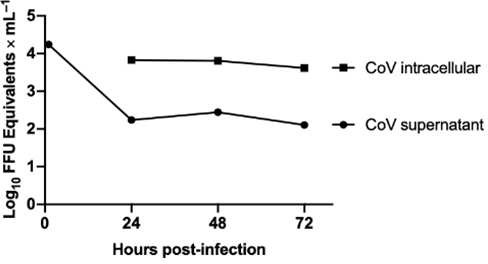


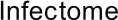
Supplementary Figure 1. A) Viral kinetic analysis in SH-SY5Y cell line. B) Viral kinetic analysis in NSC-derived neurons.


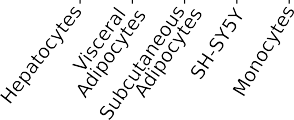

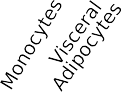

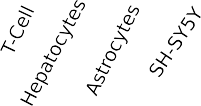

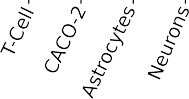

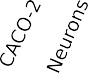

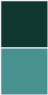

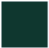

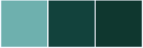

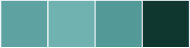

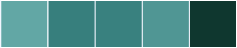

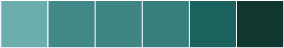

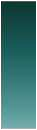

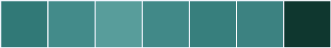

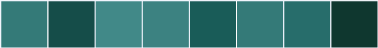

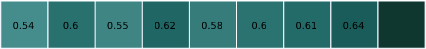

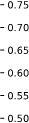

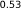

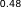

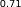

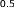

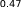

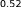

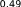

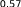

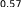

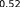

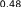

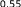

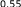

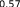

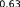

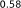

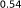

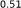

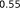

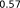

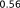

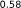

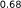

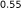

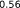

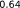

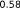

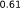

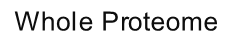

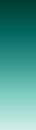

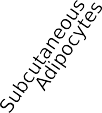


**A**

**B**

Supplementary Figure 2. Hierarchical clustering for pair-wise Pearson correlation among A) whole proteomes and B) infectomes.


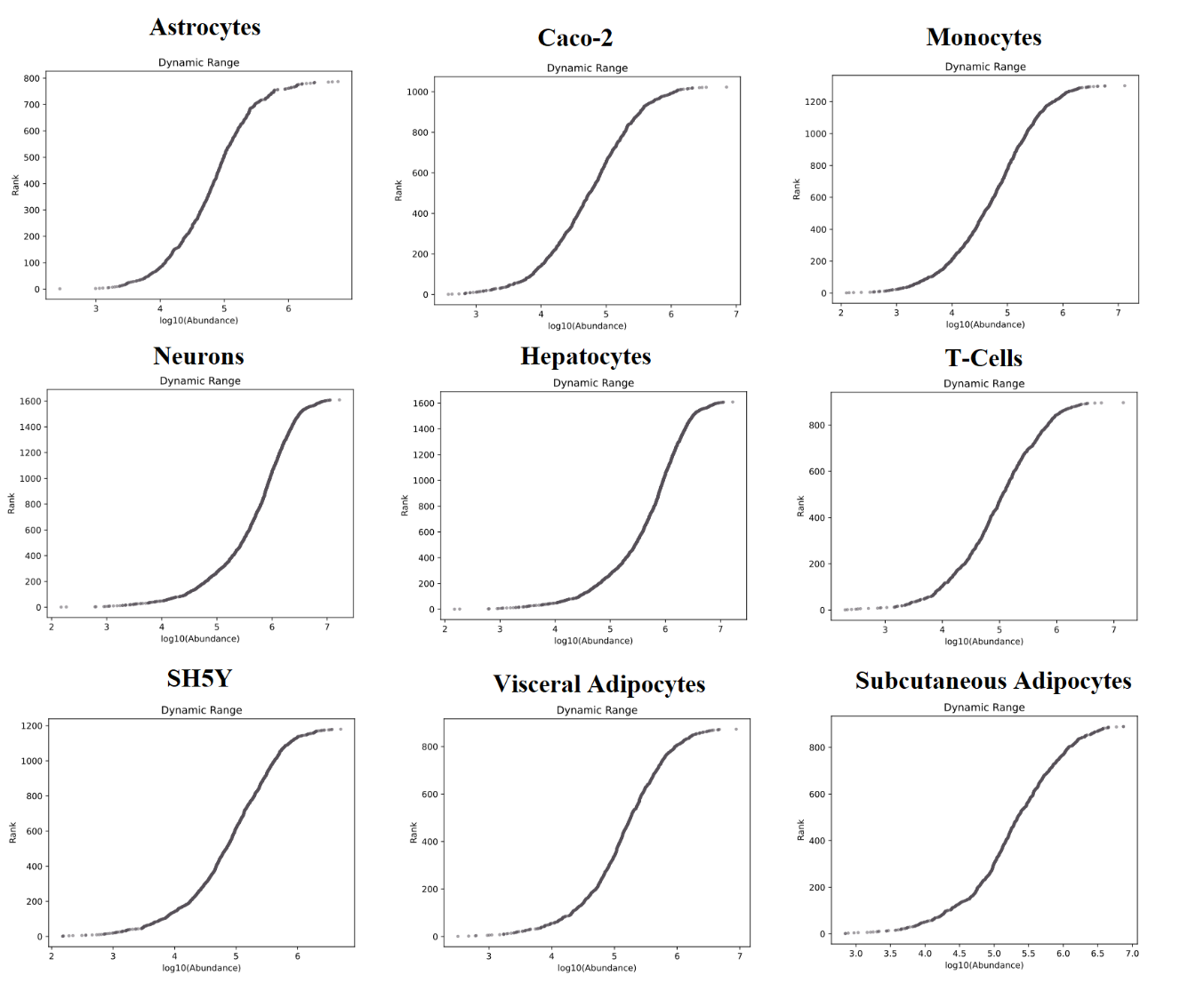


Supplementary figure 3. Dynamic range of all proteins identified for each cell type.


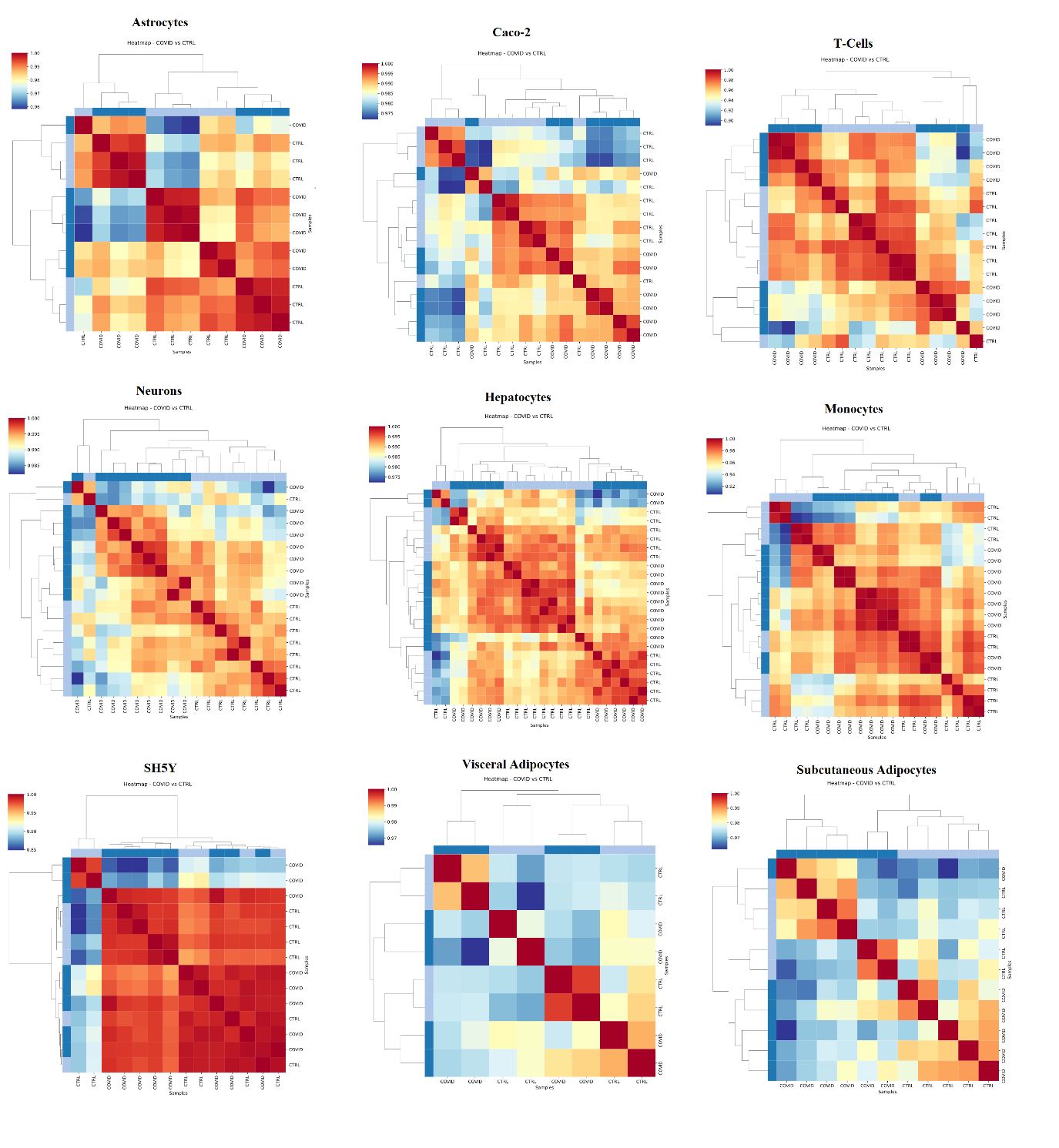


Supplementary figure 4. Pair-wise correlation analysis between all the identified proteins in all samples and hierarchical clustering analysis for the correlation matrix.


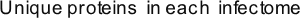


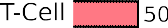

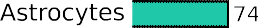

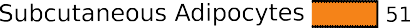

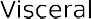

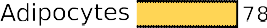

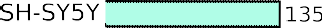

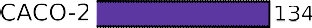

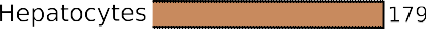

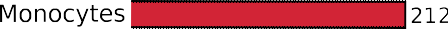

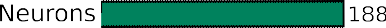

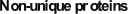

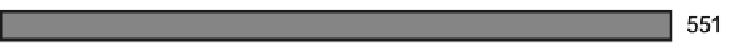


Supplementary Figure 5. Number of unique proteins differentially regulated in each infectome. Although no protein was found in all infectomes, 551 proteins were differentially regulated in more than one cell type.


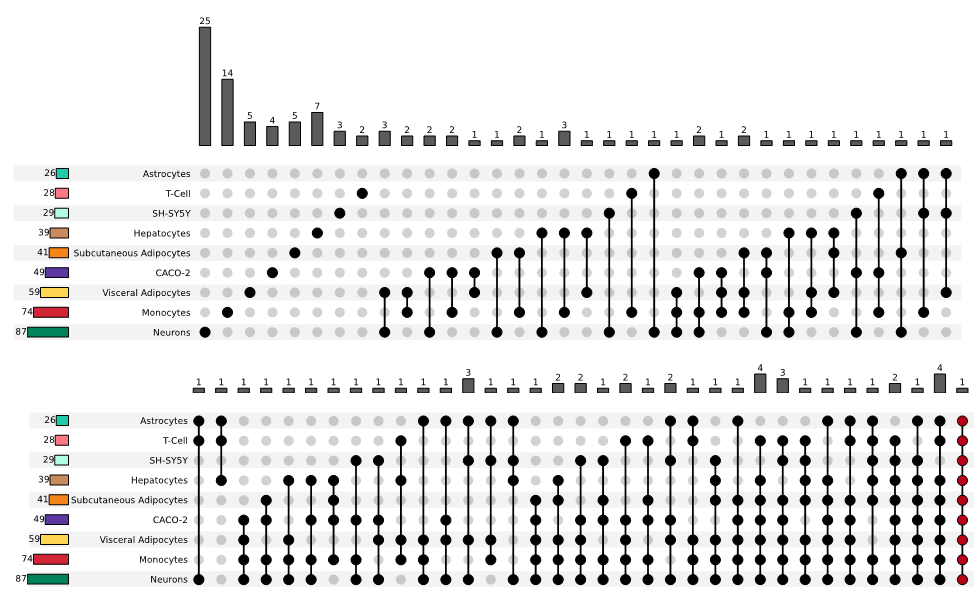


Supplementary Figure 6. Upset plot showing the number of overlapping pathways among infectomes. Vertical bars indicate the number of proteins found exclusively in groups marked by the respective connected dots. Horizontal bars indicate the total number of pathways found in each infectome.


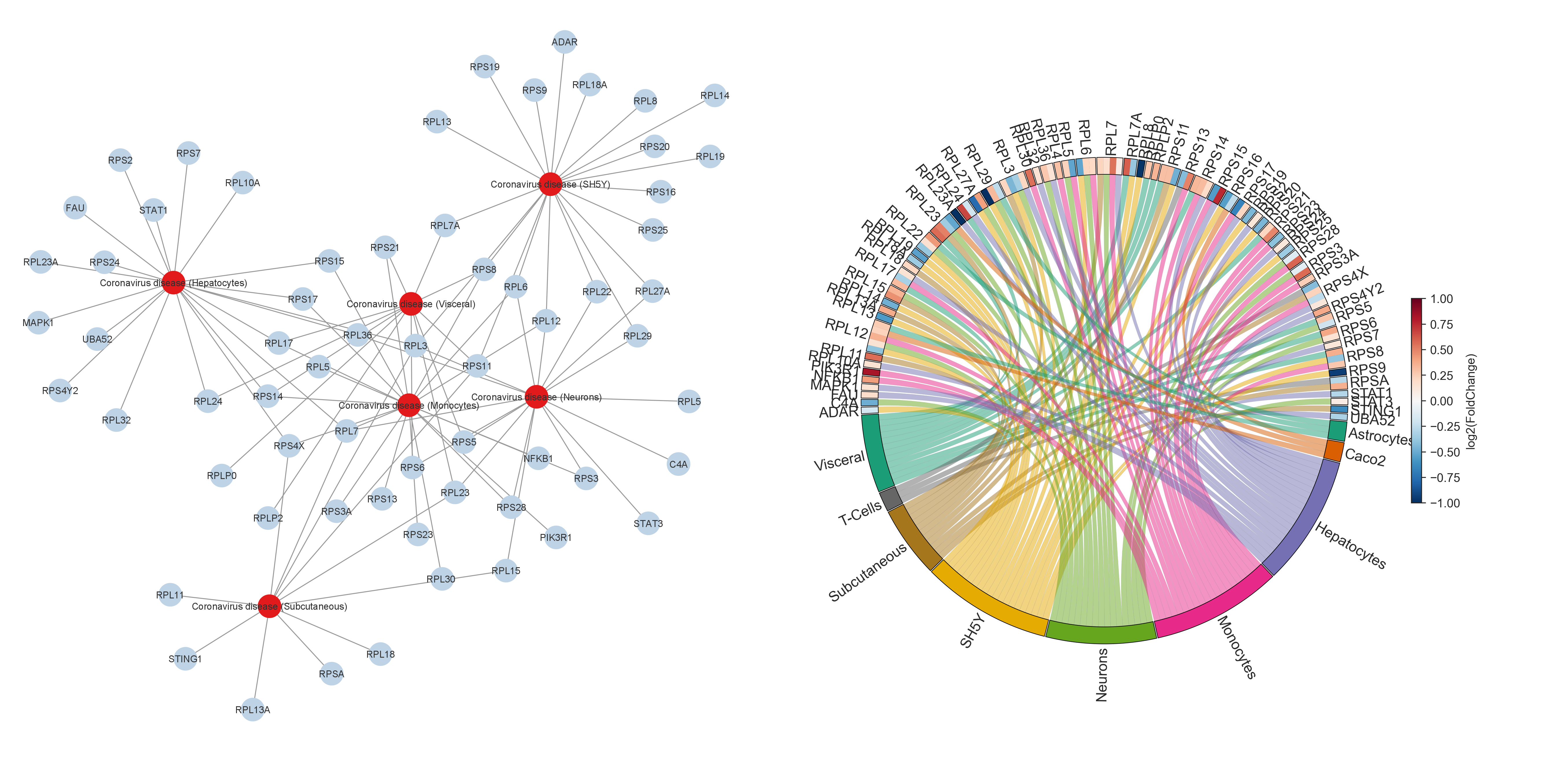


**B**

**A**

Supplementary figure 7. A -Protein-protein interactions off all proteins enriched for Coronavirus disease B- Cord diagram correlating all the proteins present in the Coronavirus disease pathway with the cell type origin.


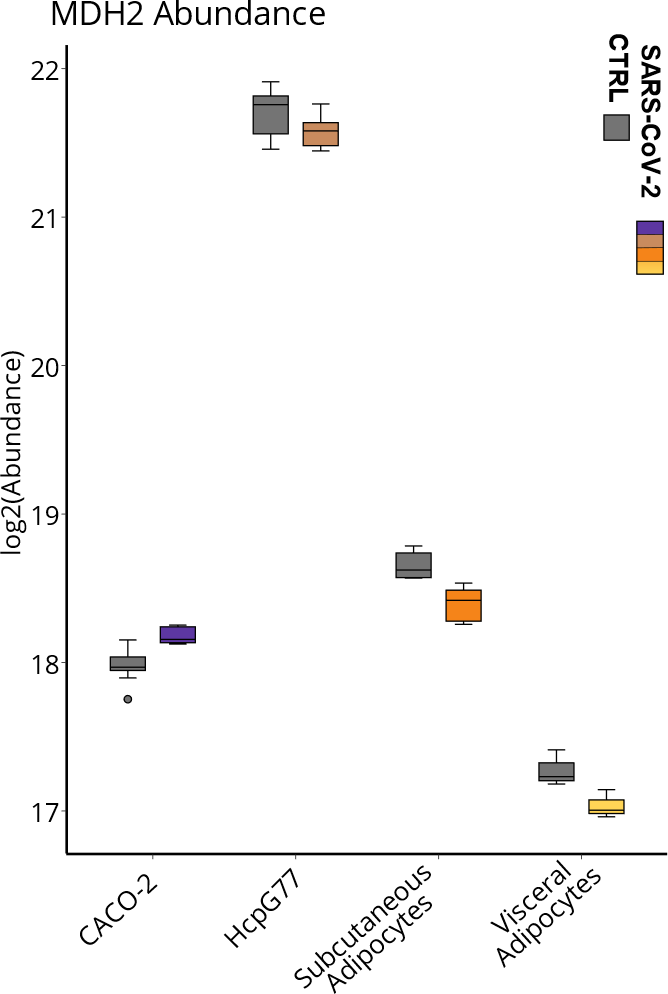


Supplementary Figure 8. Boxplot of MDH2 abundance for control (CTRL) and SARS-CoV-2-infected cells.


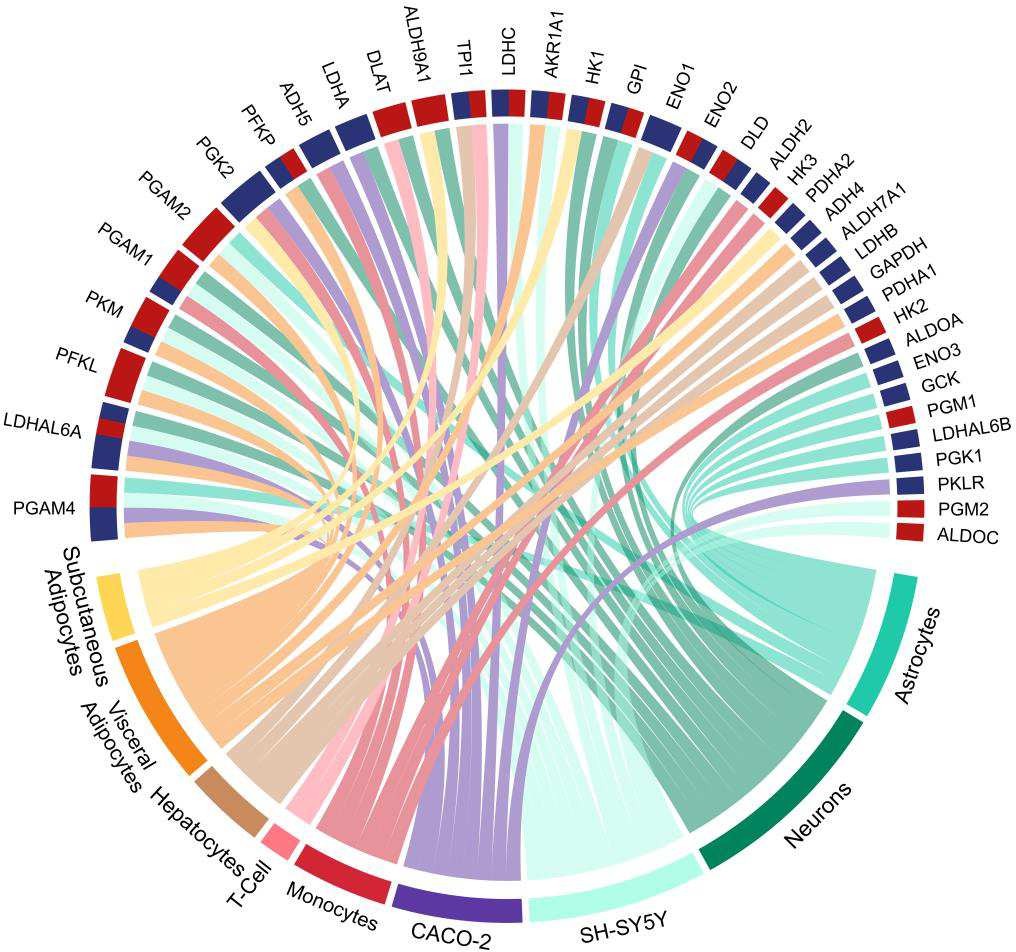


Supplementary Figure 9. Proteins found in all SARS-CoV-2 infectomes that are related to energy metabolism. Red indicates upregulation; blue indicates downregulation.

Supplementary table 1. All Proteins identified in all the different cell types.
